# Use of the Griffiths mental development scale-Chinese in the assessment of children with autism spectrum disorder and global developmental delay/intellectual disability

**DOI:** 10.1097/MD.0000000000025407

**Published:** 2021-04-02

**Authors:** Hui Wang, Yu Du, Zhenghuan Mao, Yueping Che, Haifeng Li, Li Ding, Huiying Jin

**Affiliations:** Department of Pediatric Rehabilitation, The Children's Hospital, Zhejiang University School Of Medicine, National Clinical Research Center For Child Health, Hangzhou, Zhejiang, China.

**Keywords:** autism spectrum disorder, children, global developmental delay, Griffiths mental development scale-Chinese, intellectual disability, language disorder

## Abstract

The Griffiths Mental Development Scale-Chinese (GDS-C) is used in China to assess the development of children from birth to 8 years of age. Language disorders are a common symptom of autism spectrum disorder (ASD) and global developmental delay (GDD)/intellectual disability (ID). There is a need to identify distinct clinical characteristics in children suspected of having these 2 disorders, mainly presenting as language disorders. Here, we aimed to use the GDS-C to evaluate children presenting with language problems to identify characteristics that distinguish ASD and GDD/ID. Children with language problems were recruited between August 2018 and December 2019. A total of 150 children aged 25 to 95.2 months were enrolled (50 in the ASD group, 50 in the GDD/ID group, and 50 in the typical group). Each group was subdivided by age as follows: 24–36 months, >36–60 months, and >60–96 months. Developmental characteristics assessed using the GDS-C were analyzed and compared. Both, children with ASD and GDD/ID presented with a lower developmental level than typical children in all six subscales of the GDS-C. No significant differences were observed in the six subscale scores between the ASD and GDD/ID groups, except for the practical reasoning subscale score in the >36 to 60 months subgroups, which was significantly lower in the GDD/ID group than in the ASD group. The developmental imbalance of subscales within the ASD and GDD/ID groups identified troughs in the personal-social, language, and practical reasoning areas in children with ASD and in the language and practical reasoning areas in children with GDD/ID relative to typical children. The GDS-C is a useful, comprehensive tool for the assessment of the developmental state of children with ASD and GDD/ID. Characteristics of practical reasoning subscale help diagnose autism in >36 to 60 months old children.

## 1 Introduction

Autism spectrum disorder (ASD) is a neurodevelopmental disorder characterized by impaired social and communication abilities and restricted or repetitive behaviors.^[1,2]^ Inherent in the core symptoms of ASD are differences in the use of verbal and nonverbal communication for social interactions. Patients with ASD may also have speech and language disorders, intellectual disabilities, learning disabilities, or other disorders. These conditions may affect the presentation of ASD symptoms and may influence the social and functional impairment of the individual in different ways at different ages.^[3]^ Language impairment remains a major characteristic, in addition to the core deficits of ASD. Delay in learning language may also be an early concern for many children who are later diagnosed with ASD.^[3]^ However, diagnostic difficulties can arise due to the commonality of symptoms between ASD and other conditions. Global developmental delay (GDD)/intellectual disability (ID) implies deficits in cognitive and social adaptation during the developmental stages of childhood. GDD is limited to children aged <5 years and is defined as a significant delay in two or more developmental domains, including activities of daily living and motor, cognitive, speech/language, and personal/social skills. Intellectual disability (ID) affects children aged >5 years.^[1]^ Intellectual disability is characterized by significant limitations in both intellectual functioning and adaptive behavior, expressed as problems in conceptual, social, and practical adaptive skills.^[4]^ Repetitive behaviors are also observed in children with GDD/ID when these individuals do not develop language or sign language skills. Therefore, language disorders are a group of symptoms that have characteristics similar to children with ASD and GDD/ID, making it substantially challenging in identifying and implementing targeted interventions for children with ASD or GDD/ID exhibiting significant language impairment.

The Griffiths Mental Development Scale was originally developed by Ruth Griffiths in the United Kingdom in 1954, and the main motivation for its development was the need for the early detection of developmental delays in children.^[5,6]^ Because Griffiths believed that speech is a “unique human intellectual task,”^[7]^ the Griffiths scale includes many more speech items than previously published assessment tools for children with speech problems. In addition to assessing the cognitive and perceptual skills of children, the Griffiths scale provides a comprehensive developmental profile across six separate subscales for children aged ≥2 years: locomotor (A), personal and social skills (B), hearing and language (C), hand-eye coordination (D), performance (E), and practical reasoning (F).^[8]^ The practical reasoning subscale was designed to provide a more comprehensive assessment of the emerging problem solving and logical reasoning skills of young children.

The Griffiths Mental Development Scale-Chinese (GDS-C) has been adapted to assess the development of Chinese children after completing the revision of China norm research in seven cities between 2009 and 2013. It displays good reliability and validity.^[9]^ There is little research that can help identify the developmental characteristics in children with neurodevelopmental disabilities which tend to have several clinical symptoms in common, using the assessment scale such as the GDS-C. In this study, we aimed to use the GDS-C to evaluate children with language problems as the main factor in an attempt to identify developmental characteristics that can distinguish ASD and GDD/ID.

## 2 Patients and methods

### 2.1 Subjects

Children aged between 2 and 8 years who visited the Department of Pediatric Rehabilitation, The Children's Hospital, Zhejiang University School of Medicine because of language problems were recruited between August 2018 and December 2019. The inclusion criteria were: children with symptoms of social communication/interaction or developmental delays in 2 or more domains including language disorder. The exclusion criteria were:

(1) uncontrolled epileptic seizures;
(2) visual or hearing impairment;
(3) history of central nervous system infection or traumatic brain injuries;
(4) neuromuscular disease such as cerebral palsy, muscular dystrophy, etc.

A total of 397 children were enrolled based on these criteria. The children were assigned to the ASD group and GDD/ID group according to their diagnosis. Children initially diagnosed with ASD by the third author using the DSM-V^[1,3]^ were subsequently re-evaluated by another independent child psychiatrist and were found to fulfill the criteria for ASD. Similarly, all children in the GDD/ID group diagnosed by the third author using the DSM-V^[1]^ were subsequently re-evaluated by another independent child psychiatrist. Healthy children with typical development were recruited as volunteers for a GDS-C training mission and served as a control group. They did not have any language problems. Each group comprised of 50 children. The groups were subdivided into a 24 to 36 months group (children aged 24 months to 36 months), a >36–60 months group (children older than 36 months to 60 months), and a >60–96 months group (children older than 60 months to 96 months). Within the GDD/ID group, the 24 to 36 months subgroup and the > 36–60 months subgroup were assigned to the GDD group, and the >60–96 months subgroup was assigned to the ID group. This study was approved by the Ethics Committee of the Children's Hospital of Zhejiang University School of Medicine (2020-IRB-040). The parents or guardians of all included children provided written informed consent.

### 2.2 Instruments

The GDS was modified and adapted as GDS-C for use in Chinese children due to differences between the Chinese and British developmental curves, providing reliable developmental curves for Chinese children up to 8 years of age.^[9]^ According to clinical research, the GDS-C effectively evaluates motor function, learning difficulties, congenital mental development status and developmental disorder, vision problems, autism, degree of premature birth, and social/emotional development skills in Chinese children. In this study, children with language problems were assessed using the GDS-C by 2 assessors who were both registered Griffiths scale users and were experienced in conducting psychological tests. Each assessor viewed the video of a child being tested and then submitted a report of their assessment to ensure that the overall scoring agreement was within two items per scale. Six subscales (locomotor [A], personal-social [B], language [C], hand-eye coordination [D], performance [E], and practical reasoning [F]) were separately administered and scored according to a standardized procedure. The raw scores of the six subscales were converted to the corresponding percentiles and functional age standard by the Chinese norm. The functional age recorded in each subscale was obtained through ascribed computations. Developmental quotients (DQs) were determined for each subscale by dividing the functional age of the child by his chronological age at the time of testing. The results are reported as DQs with a mean of 100 and a standard deviation (SD) of 15.

DQ = functional age/chronologic age × 100

### 2.3 Statistical analysis

All statistical analyses were performed using IBM SPSS Statistics for Windows, version 20.0 (IBM, Armonk, NY, USA). A normality test was conducted for continuous data. Continuous data with a normal distribution are presented as the mean ± SD and were analyzed using Student's *t* test and ANOVA with the least significant difference post hoc test for comparisons among three or more groups. Continuous data with a skewed distribution are described as medians (ranges) and were analyzed using the Mann-Whitney test for comparison between the 2 groups. Wilcoxon test was used for comparing the status before and after treatment, and the Kruskal-Wallis test with the Mann-Whitney post hoc test was used for comparing three or more groups. *P* values of <.05 were considered statistically significant.

## 3 Results

### 3.1 Comparisons among three groups

**Figure 1:**
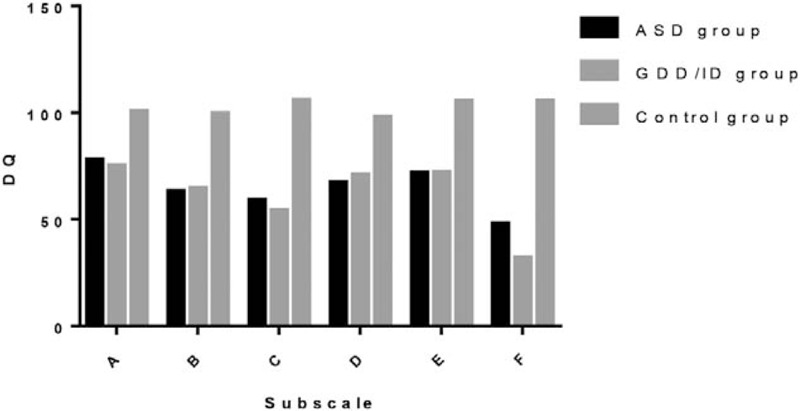
Comparison of developmental quotient scores for the Griffiths Mental Development Scale-Chinese subscales among the three groups. A = locomotor, ASD = autism spectrum disorder, B = personal-social, C = language, D = hand-eye coordination, DQ = developmental quotient, E = performance, F = practical reasoning, GDD/ID = global developmental delay/intellectual disability.

As shown in Table 1, the mean age, number of children in each subgroup, and sex ratio did not significantly differ among the groups. The DQ scores for each subscale were significantly lower in both the ASD and GDD/ID groups than in the control group (all *P* < .05), while no significant differences were observed in the DQ scores between the ASD and GDD/ID groups (all *P* > .05). The results of further analyses are shown in Figure 1.

**Table 1:** Comparison of the parameters among the three groups.

	ASD group (n = 50)	GDD/ID group (n = 50)	Control group (n = 50)	*P*^∗^	*P*
Age (months)	54.13 ± 19.56	50.25 ± 17.31	51.47 ± 17.93	.296	.556
24–36 months, n	11	11	11		
>36–60 months, n	21	23	23		
>60–96 months, n	18	16	16		
M:F	44:6	36:14	32:18		
GDS-C subscale scores					
Locomotor (A)	78.30 (65.40–92.33)	74.40 (60.03–92.43)	100.00 (91.5–110.18)	1.000	<.001
Personal-social (B)	60.90 (52.30–73.58)	64.95 (52.83–75.88)	99.00 (92.98–106.38)	1.000	<.001
Language (C)	58.6 (39.63–76.25)	56.35 (39.03–67.45)	101.35 (91.30–117.98)	1.000	<.001
Hand-eye coordination (D)	67.80 (57.30–76.53)	71.90 (55.20–85.45)	97.90 (90.08–104.83)	.930	<.001
Performance (E)	66.70 (55.78–88.40)	71.15 (55.30–91.78)	102.00 (95.20–112.10)	1.000	<.001
Practical reasoning (F)	53.75 (0.00–79.93)	0.00 (0.00–64.68)	104.90 (94.55–117.50)	.420	<.001

Data are presented as medians and interquartile ranges. ASD = autism spectrum disorder, F = female, GDD/ID = global developmental delay/intellectual disability, GDS-C = Griffiths Mental Development Scale-Chinese, M = male. *P*^∗^: Comparison between the ASD group and GDD/ID group. *P*: Comparison among 3 groups.

**Figure 2:**
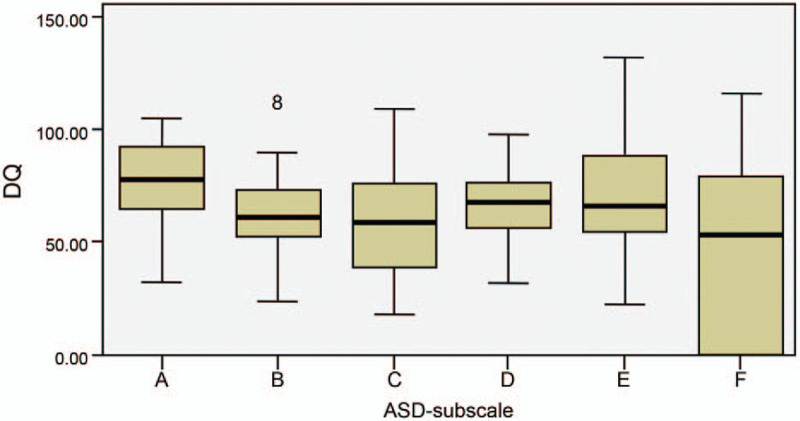
Comparison of developmental quotient scores for the Griffiths Mental Development Scale-Chinese subscales in the autism spectrum disorder group. A = locomotor, ASD = autism spectrum disorder, B = personal-social, C = language, D = hand-eye coordination, DQ = developmental quotient, E = performance, F = practical reasoning.

In the ASD group, the DQ scores of the B, C, and F subscales were significantly lower than those of the A subscale (*P* = .002, < .001, and < .001, respectively). No significant differences were observed among the A, D, and E subscales (*P* = .131, .794, and 1.000, respectively) (Table 2 and Fig. 2).

**Table 2:** Comparison of developmental quotient scores for the Griffiths Mental Development Scale-Chinese subscales in the autism spectrum disorder group.

Subscale	B^∗^	C^∗^	D	E	F^∗^
A	0.002^∗^	<0.001^∗^	0.131	0.794	<0.001^∗^
B		1.000	1.000	0.896	1.000
C			1.000	0.274	1.000
D				1.000	0.551
E					0.083

A = locomotor, B = personal-social, C = language, D = hand-eye coordination, E = performance, F = practical reasoning.

∗ *P* < .05.

**Figure 3:**
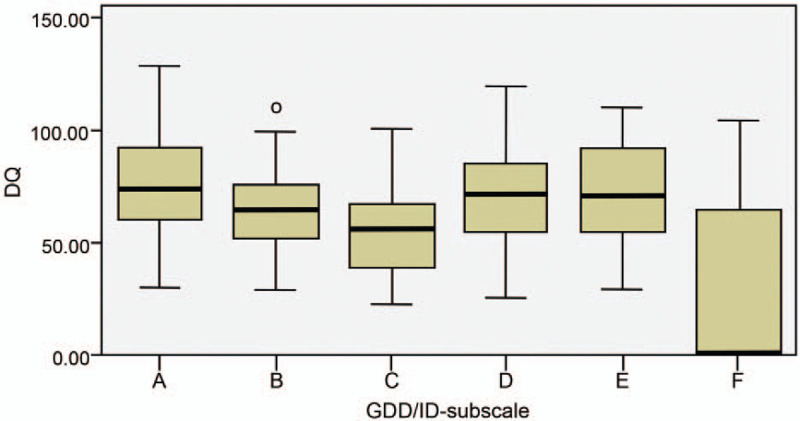
Comparison of developmental quotient scores for the Griffiths Mental Development Scale-Chinese subscales in the global developmental delay/intellectual disability group. A = locomotor, B = personal-social, C = language, D = hand-eye coordination, DQ = developmental quotient, E = performance, F = practical reasoning, GDD/ID = global developmental delay/intellectual disability.

In the GDD/ID group, the DQ score of the C subscale was significantly lower than those of the A, D, and E subscales (*P* < .001, .008, and .003, respectively). The DQ score of the F subscale was significantly lower than that of the A, B, D, and E subscales (*P* < .001, .002, < .001, and < .001, respectively). No significant differences were observed among the A, B, D, and E subscales (*P* = .436, 1.000, 1.000, 1.000, 1.000, and 1.000, respectively) (Table 3 and Fig. 3).

**Table 3:** Comparison of developmental quotient scores for the Griffiths Mental Development Scale-Chinese subscales in the global developmental delay/intellectual disability group.

Subscale	B	C^∗^	D	E	F^∗^
A	0.436	<0.001^∗^	1.000	1.000	<0.001^∗^
B		0.477	1.000	1.000	0.002^∗^
C			0.008^∗^	0.003^∗^	1.000
D				1.000	<0.001^∗^
E					<0.001^∗^

A = locomotor, B = personal-social, C = language, D = hand-eye coordination, E = performance, F = practical reasoning.

∗ *P* < .05.

### 3.2 Comparison between the ASD group and GDD group at 24 to 36 months, >36 to 60 months, and ID group at >60 to 96 months

The comparisons of the DQ scores in the ASD group and GDD group at 24 to 36 months, >36 to 60 months, and ID group at >60 to 96 months are shown in Table 4. In the GDD group at 24 to 36 months and ID group at >60 to 96 months subgroups, the scores for each subscale were similar between the ASD and GDD/ID groups, with no significant differences (*P*> .05). At >36 to 60 months GDD group, the DQ scores for the subscales were similar between the two groups, with no significant differences, except the F subscale, which was significantly lower in the GDD group than in the ASD group (*P* = .019).

**Table 4:** Comparison of developmental quotient scores for the Griffiths Mental Development Scale-Chinese subscales between the three age subgroups of the autism spectrum disorder group and global developmental delay/intellectual disability group.

	24–36 months			>36–60 months			>60–96 months		
Variables	ASD group (n = 11)	GDD group (n = 11)	*P*	ASD group (n = 21)	GDD group (n = 23)	*P*	ASD group (n = 18)	ID group (n = 16)	*P*
Age (months)	30.01 ± 3.18	30.82 ± 2.87	.538	48.13 ± 7.88	44.08 ± 6.78	.074	75.88 ± 10.09	72.48 ± 6.58	.260
Locomotor (A)	78.30 (74.00–90.30)	71.70 (60.70–92.50)	.393	91.00 (72.25–96.65)	83.30 (69.60–96.20)	.518	65.10 (60.00–79.18)	64.80 (50.08–74.50)	.546
Personal-social (B)	52.00 (48.30–64.30)	62.90 (53.50–68.90)	.148	67.10 (53.10–78.50)	65.70 (45.20–77.30)	.751	60.90 (57.23–68.33)	66.80 (49.90–76.70)	.438
Language (C)	58.90 (33.30–68.10)	47.50 (42.00–61.70)	.870	69.00 (44.95–79.10)	57.50 (30.40–71.10)	.209	52.90 (46.48–71.18)	58.15 (45.28–65.63)	.863
Hand-eye coordination (D)	75.00 (62.50–82.10)	63.50 (53.70–83.90)	.308	67.40 (54.30–74.55)	73.60 (57.90–89.30)	.204	63.10 (50.25–77.60)	78.50 (49.43–87.20)	.581
Performance (E)	60.00 (58.90–89.30)	71.00 (44.90–90.10)	.974	75.90 (57.25–85.30)	71.20 (55.80–90.40)	.991	61.50 (53.28–92.99)	71.85 (58.95–93.35)	.593
Practical reasoning (F)	0.00 (0.00–0.00)	0.00 (0.00–0.00)	.475	66.30 (0.00–81.35)	0.00 (0.00–51.30)	.019^∗^	62.45 (43.18–62.45)	64.20 (45.8–80.23)	.945

Data are presented as medians and interquartile ranges. ASD = autism spectrum disorder, GDD/ID = global developmental delay/intellectual disability.

## 4 Discussion

Using the GDS-C, we reviewed the developmental characteristics of children aged 2 to 8 years with ASD and GDD/ID who presented with language impairment. In this study, we observed a comprehensive developmental delay in both the ASD group and GDD/ID group, as assessed using the GDS-C, including locomotor (A), personal-social (B), language (C), hand-eye coordination (D), performance (E), and practical reasoning (F), with significant differences relative to the control group. Thus, children with ASD and GDD/ID were thought to have overall developmental delays.

The dysfunction observed in children with ASD encompasses several interconnected domains, including cognitive, language, social and emotional, and adaptive behavior domains. However, many studies have reported the presence of motor and sensory difficulties in children with autism during early development. ^[10–14]^ The developmental delay of the motor function includes gross and fine motor skills.^[15]^ These studies reported substantial difficulties in balance, postural stability, movement speed, and strength in the ASD group.^[16–19]^ Similarly, a vulnerability in motor function during early development positively correlates with the appearance of clinical symptoms in children with ASD.^[20]^ These children experience particular difficulty coordinating movements that involve both sides of their body or both arms and legs.^[21]^

Children with GDD/ID have intellectual and adaptive deficits and present with delays in attaining developmental milestones at the expected age, which implies deficits in learning and adaptation.^[1,22]^ In addition, a language barrier was the main characteristic of children in the GDD/ID group in our study. In this group, significant delays were observed in two or more developmental domains, including language and cognitive domains, suggesting that these delays are most noticeable. Further, the ability of these children to learn other behaviors, including sports, hand-eye coordination, and visual-spatial and reasoning skills, may also be affected.

Although no significant difference was observed between the ASD group and the GDD/ID group, both groups recorded the lowest DQ scores for the language subscale and the practical reasoning subscale and high scores for the locomotor subscale, with significant differences relative to the other subscales. In addition, in the ASD group, the DQ score of the personal-social subscale was significantly lower than that of the locomotor subscale but did not significantly differ from the practical reasoning subscale score, indicating a significant impairment in personal-social ability. In the GDD/ID group, the impairments in language and practical reasoning were dominant, and the DQ score of the practical reasoning subscale significantly differed from the locomotor, personal-social, hand-eye coordination, and performance subscales, indicating that the impairment was more apparent in practical reasoning than in other areas. Our results obtained from children with ASD were similar to those from a study on 70 children and adolescents who were assessed using the Griffiths Mental Development Scale, i.e., a characteristic profile emerged with peaks in motor and visuospatial domains and troughs in verbal and practical reasoning areas.^[23]^ Impairments in expressive and impressive verbal functioning are common among children with autism because of the impairment in communication, the most important symptom of autism. Thus, practical reasoning skills are also impaired.

Children with ASD may exhibit less dyskinesia compared to typical children. In our study, the majority of children with ASD were able to perform motor tasks and only exhibited slightly reduced motor function, showing consistent qualitative differences in some of the skills.^[21]^ We also observed significant deficits in personal-social skills, which are consistent with the core symptoms of autism, namely, social communication inability. Thus, the test items of the personal-social subscale can specifically assess the core symptoms in children with ASD. In contrast, the intellectual and adaptive disabilities associated with GDD/ID involve reasoning, problem-solving, planning, abstract thinking, problem judgment, the ability to learn from guidance and experience, practical understanding, and the ability to participate socially and live independently.^[1,22,24]^ The core deficit is intellectual disability, with key components including speech comprehension, working memory, perceptual reasoning, quantitative reasoning, abstract thinking, and cognitive efficacy.^[1]^ Consistent with the results of our GDS-C assessment of children with GDD/ID, the language and practical reasoning skills were significantly impaired, with particularly high impact on practical reasoning skills.

Furthermore, we compared the subgroups stratified by age between the ASD group and GDD group: 24 to 36 months, >36 to 60 months, and ID group: >60–96 months. We observed a significant difference (*P* = .019) in the practical reasoning subscale at >36–60 months between the ASD group and GDD group, but not at 24 to 36 months GDD group or >60 to 96 months ID group (*P* = .474 and 0.945, respectively). Regarding the possible causes, in the 24 to 36 months subgroup, the F subscale is suitable for assessing the development of children aged >2 years. We were unable to obtain a score for the F subscale in children aged 24–36 months in either group due to the developmental delay, resulting in non-significant differences between the two groups. In the >60 to 96 months subgroup, the 99th percentile curve of the F subscale of the GDS-C plateaued soon after 4.5 years of age,^[9]^ suggesting the limitation of assessing children aged >4.5 years. Thus, no significant differences were observed between the 2 groups.

Some study limitations may also need to be considered. The smaller numbers of cases in the 24 to 36 months and >60 to 96 months subgroups may have affected the results of our study. Further studies should include a greater number of participants in the relevant subgroups. Future research should also consider the use of different samples of GDD and ID groups because of age-related differences between these two conditions.

In conclusion, we used GDS-C to assess children with ASD and GDD/ID, and identified comprehensive developmental delay with troughs in personal-social, language, and practical reasoning skills in children with ASD and language and practical reasoning skills in children with GDD/ID. The practical reasoning subscale score is useful for distinguishing children with ASD and GDD/ID at the age of >36 to 60 months. GDS-C is valuable as a reference tool for determining a differential diagnosis in the clinic and for the selection of interventions and treatment for ASD and GDD/ID.

## Acknowledgments

The authors thank all of the doctors, therapists, nurses, and other staff members at the Department of Pediatric Rehabilitation, Children's Hospital, Zhejiang University School of Medicine, National Clinical Research Center for Child Health, who kindly provided their cooperation in this study. to the authors also thank Editage (www.editage.com) for English language editing.

## Author contributions

**Data curation:** Hui Wang, Zhenghuan Mao, Yueping Che, Huiying Jin.

**Funding acquisition:** Hui Wang, Yueping Che, Huiying Jin.

**Investigation:** Yu Du, Li Ding.

**Methodology:** Hui Wang, Zhenghuan Mao, Haifeng Li.

**Supervision:** Yu Du, Haifeng Li, Li Ding.

**Validation:** Yueping Che, Haifeng Li.

**Writing – original draft:** Hui Wang, Yu Du, Zhenghuan Mao.

**Writing – review & editing:** Hui Wang.
